# When Size Goes Inside: Visceromegaly in Bodybuilders Is Not Attributed to High-Protein Intake but More Likely Associated with the Use of Appearance- and Performance-Enhancing Drugs

**DOI:** 10.1249/MSS.0000000000004011

**Published:** 2026-04-27

**Authors:** CAS J. FUCHS, BENTE BRAUWERS, JORN TROMMELEN, SERHAT YILDIZ, PER WIDHOLM, OLOF DAHLQVIST LEINHARD, JEANINE J. PROMPERS, LUC J. C. VAN LOON

**Affiliations:** 1 Department of Human Biology, Institute of Nutrition and Translational Research in Metabolism (NUTRIM), Maastricht University Medical Centre^+^, Maastricht, THE NETHERLANDS; 2 AMRA Medical AB, Linköping, SWEDEN

**Keywords:** ANDROGENIC-ANABOLIC STEROIDS, BUBBLE GUT, HORMONES, LEAN MASS, MAGNETIC RESONANCE IMAGING, MUSCLE MASS, ORGAN VOLUME, PALUMBOISM, RESISTANCE EXERCISE TRAINING

## Abstract

**Background:**

Bodybuilders use progressive resistance exercise training and high-protein-intake diets to develop muscular physiques. Some also use appearance- and performance-enhancing drugs (APED) to further enhance their physique. It has been speculated that a high-protein intake and APED use may induce visceral organ hypertrophy. Therefore, the primary objective of this study was to assess visceral organ volumes in natural and APED-using male bodybuilders and to compare these with recreationally active males (CON).

**Methods:**

Magnetic resonance imaging (MRI) was applied to assess skeletal muscle and visceral organ volume in 15 CON (age: 29 ± 6 y), 15 competitive male natural bodybuilders (BB-NAT; age: 30 ± 5 y), and 15 competitive male APED-using bodybuilders (BB-USE; age: 31 ± 4 y). In addition, dual-energy X-ray absorptiometry (DXA) was performed to assess total body lean mass. All participants reported their habitual food intake (via 7-d food diary) and, if applicable, their use of APED.

**Results:**

Habitual protein intake was much higher in the bodybuilders (BB-NAT: 2.5 ± 0.6 g/kg body mass (BM)/d; BB-USE: 2.9 ± 0.4 g/kg BM/d) compared with CON (1.4 ± 0.3 g/kg BM/d; *P* < 0.001), with no differences between both bodybuilder groups (*P* = 0.109). Total lean BM was significantly different across all groups and was greater in BB-USE (87.8 ± 8.0 kg) versus BB-NAT (72.7 ± 6.4 kg) versus CON (62.8 ± 4.8 kg; *P* < 0.001). Muscle volumes were also different between groups, with average thigh muscle volumes of 7.1 ± 1.0, 5.8 ± 0.7, and 4.5 ± 0.4 L in BB-USE, BB-NAT, and CON, respectively (*P* < 0.001). All visceral organ volumes were greater in BB-USE compared with BB-NAT and CON (*P* < 0.05), with no differences between BB-NAT and CON (*P* > 0.05). Heart volume averaged 1.20 ± 0.15, 0.89 ± 0.14, and 0.83 ± 0.15 L in BB-USE, BB-NAT, and CON, respectively. Liver volume averaged 2.57 ± 0.39, 1.82 ± 0.20, and 1.79 ± 0.21 L, respectively.

**Conclusions:**

Despite higher habitual protein intake and greater lean BM, natural bodybuilders do not show greater visceral organ volumes compared to age-matched controls. Bodybuilders using APED show substantially greater lean mass, muscle mass, as well as greater visceral organ volumes, compared with natural bodybuilders or age-matched controls. We conclude that visceromegaly in bodybuilders cannot be attributed to a high-protein intake but is secondary to the use of APED.

Competitive bodybuilders perform structured resistance-type exercise training to develop a muscular, symmetrical, and well-proportioned physique. Additionally, bodybuilders typically adopt a high-protein diet with the aim to further stimulate muscle protein synthesis rates and support muscle hypertrophy. Previous research has reported habitual protein intakes ranging from 2 to well over 4 g of protein per kg body mass (kgBM) per day in competitive bodybuilders (1). Such protein intake levels (substantially) exceed current protein intake recommendations (2,3) as well as average protein intake levels observed in many other athletic populations (~1.5 g/kgBM/d) and recreationally resistance-trained men (~1.6 g/kgBM/d) (4,5). The proposed benefits of such high-protein intakes in bodybuilders to further augment muscle hypertrophy remain speculative at best.

Beyond its role in skeletal muscle anabolism, there is speculation that sustained high-protein intake may also stimulate organ tissue protein synthesis and, as such, increase the size of visceral organs (6). Furthermore, the metabolic processing of large amounts of food, in particular protein, may impose greater demands on organs involved in protein/amino acid metabolism and nitrogen excretion, such as the liver and kidneys. Several studies in both animals and humans have reported that high-protein diets can induce hypertrophy of these organs (7–12), although findings across the literature remain inconsistent (13). Animal research further suggests that long-term resistance exercise training may mitigate hepatic and renal hypertrophy associated with high-protein feeding (8,11,12). In contrast, human studies comparing resistance-trained individuals with their untrained counterparts have observed greater liver and kidney volumes in the trained groups, which has been speculated to be caused at least partly by a high-protein intake level (14,15). Notably, regardless of potential effects on organ size, high-protein intakes (at least up to ~3.3 g/kg/d) do not appear to adversely affect liver or kidney function in healthy individuals (16–18). In short, it remains to be established whether the greater total lean BM observed in competitive natural bodybuilders, who typically combine structured resistance training with a high-protein intake, is partly attributed to an increase in visceral organ volume.

In addition to structured resistance exercise training and high-protein-intake diets, many competitive bodybuilders also use appearance- and performance-enhancing drugs (APED) such as anabolic-androgenic steroids and other nonsteroidal hormones (e.g., insulin, human growth hormone, thyroid hormones, and clenbuterol) to further augment muscle hypertrophy and reduce fat mass (19,20). Although these substances may support greater increases in skeletal muscle mass (21–23), their use has also been linked to abnormal enlargement and protrusion of the abdominal region (24). The latter phenomenon is commonly described in popular media by terms such as “Palumboism,” “bubble gut,” or “steroid gut.” Moreover, beyond the abdominal cavity, serious concerns have been raised regarding reports of increased cardiac size in bodybuilders using APED (25,26). Hypertrophy of visceral organs, particularly the heart, may lead to several health complications and may elevate the risk of premature death in this population (24,27–30). To date, there are no quantitative data comparing visceral organ volumes in bodybuilders using APED versus bodybuilders or recreationally active adults not using these drugs. This paucity of data is likely owing to the legal and ethical constraints associated with studying APED use, which have largely prevented researchers from conducting controlled studies in this population. In addition, accurately assessing organ volumes requires advanced imaging techniques and data processing, which are relatively expensive and require specialized expertise.

In the present study, the primary objective was to compare visceral organ volumes (liver, heart, spleen, and kidneys) between recreationally active males (CON), competitive natural bodybuilders, and competitive bodybuilders using APED. We hypothesized that APED-using bodybuilders would show the greater visceral organ volumes, followed by natural bodybuilders, with the lowest values in recreationally active controls.

## METHODS

### Participants

Forty-five males (aged 22–41 yr) participated in this study. Fifteen CON, 15 competitive male natural bodybuilders (BB-NAT), and 15 competitive male APED-using bodybuilders (BB-USE) were recruited. All participants were deemed healthy based on their responses to a medical questionnaire, which included questions on current health status, medication use, musculoskeletal complaints, previous surgeries, chronic diseases, and relevant medical history.

Participants in the CON group were recreationally active, performing 1–3 sessions of recreational exercise per week (e.g., running, cycling, or intermittent sports), but not engaged in structured or progressive resistance exercise training. Participants in the bodybuilder groups were current or recent competitive bodybuilders, either competing in natural (drug-tested) divisions or in other (nontested) bodybuilding categories. No inclusion or exclusion criteria were applied regarding timing or the phase of participants’ most recent bodybuilding competition (e.g., pre- or postcompetition). Natural bodybuilders additionally reported no history of APED use. Bodybuilders using APED were eligible to participate if they were currently using any type(s) of APED, without restrictions on the specific substance, dose, frequency, or route of administration. All participants were informed on the nature and risks of the experiment before written informed consent was obtained. The current study was approved by the Medical Ethical Committee of Maastricht University Medical Centre (number 19-003) in accordance with the Declaration of Helsinki. This trial was registered at the former Dutch trialregister.nl, available via <https://www.onderzoekmetmensen.nl/en/trial/48513>.

### Resistance training characteristics

Participants were asked to provide detailed information about their exercise training program through a survey. Reported variables included exercise training duration (how many years of structured resistance exercise training), training frequency (days per week), total weekly training time (minutes per week), average exercise session duration (minutes per session), and the frequency with which major muscle groups were trained.

### Habitual dietary food intake

All participants completed a detailed 7-d food intake diary in the period surrounding the testing session, reflecting their habitual dietary intake. Participants received written and verbal instructions on how to record all foods, beverages, and dietary supplements consumed. Diaries were checked by a certified dietician for completeness and plausibility. Energy and macronutrient intake were calculated with the use of the Dutch Nutrients Database (NEVO-online version 2019/6.0; https://nevo-online.rivm.nl/).

### Use of appearance- and performance-enhancing drugs

In the BB-USE group, participants provided detailed information on their APED use. For privacy reasons, individual intake data are not reported. However, intakes were summarized at the group level. Reported substances covered multiple classes of anabolic-androgenic agents, including testosterone-based, dihydrotestosterone-based, and 19-nortestosterone-based compounds, as well as a range of ancillary compounds such as hormone modulators, peptide hormones, thyroid-regulating agents, and sympathomimetic substances.

### Baseline measurements

Participants arrived at the laboratory after an overnight fast and refrained from vigorous physical activity and alcohol consumption for 48 h before testing. Upon arrival, they were asked to empty their bladder and bowels. BM (kg) and height (m; Seca 799, Seca GmbH & Co., Hamburg, Germany) were measured with participants wearing minimal clothing (underwear), followed by assessment of resting blood pressure (mmHg) and heart rate using an automated device (Omron HEM-907, Omron Healthcare, Kyoto, Japan). Basal metabolic rate was measured using indirect calorimetry (Omnical, Maastricht University, Maastricht, the Netherlands). During this measurement, participants were instructed to lie down in a supine position and remain motionless for 30–35 min. Measurements were performed in a thermoneutral environment and in resting, but awakened, state. The first 10–15 min of data were discarded to eliminate the effects of acclimatization to the testing procedure. Basal metabolic rate was derived from the O_2_ consumption and CO_2_ production using the Weir formula (31). Subsequently, DXA and magnetic resonance imaging (MRI) were performed under the same overnight fasted and rested conditions (see later).

### Dual-energy X-ray absorptiometry (DXA)

Whole-body composition was measured using DXA (Hologic Discovery A; QDR Series, Marlborough, MA). The system’s software package Apex version 4.5.3, together with Hologic Physician’s Viewer (version 7.1), was used. The DXA provided measures of fat mass, bone mineral content, bone mineral density, and lean BM, which in this study refers to DXA-derived lean soft tissue mass (i.e., total BM minus fat mass and BMC) (32). All DXA scans and analyses were performed on the same system by the same trained technician. Each participant underwent scanning in a supine position with knees and hips fully extended and arms resting next to the trunk with the hands in a pronated position. In cases where bodybuilders did not fit entirely within the scan field, positioning was adjusted to ensure full coverage of the torso and legs, and one arm was mirrored using the software’s reflection mode to provide complete body composition estimates. This partial-scan reflection approach has been validated for whole-body composition assessment in broadly built individuals (33).

### Magnetic Resonance Imaging

Whole-body MRI scans were performed on a 3.0 T system (MAGNETOM Prisma Fit, Siemens Healthineers, Erlangen, Germany) using the integrated posterior spine array in combination with anterior body and peripheral coils. Participants were positioned supine and entered the magnet feet first. Images were acquired using previously published methodology for detailed whole-body muscle analysis (34). In summary, T1-weighted 3D multiecho Dixon sequences were acquired to cover the entire body from feet to neck in overlapping stacks, generating water, fat, in-phase, and opposed-phase images. The total acquisition time for the protocol ranged from ~60 to 150 min/participant, depending on body size.

The acquired Dixon images were analyzed using both semiautomatic and manual segmentation approaches. Semiautomatic segmentation was performed to assess muscle volumes using AMRA Researcher (AMRA Medical AB, Linköping, Sweden). The following muscle groups were quantified: Thigh: *quadriceps femoris, hamstrings, adductors*; Torso (chest and back): *pectoralis major, latissimus dorsi/teres major, spinal erectors (Th1–sacrum*); Shoulder and upper back: *deltoid, trapezius*; Upper arms: *biceps brachii, triceps brachii*. In addition, manual volumetric analysis was performed using ITK-SNAP software (35). Organ volumes of the liver, heart, spleen, and kidneys were delineated manually. All assessors were blinded to participant identity.

### Statistical analysis

Sample size estimation was based on previously reported differences in liver mass between resistance-trained and -untrained males (15). Using the reported means and SDs, an effect size of *f* ≈ 0.55 was estimated. An a priori power analysis in G*Power (one-way ANOVA, three groups, α = 0.05, power = 0.80) indicated that a minimum sample size of 36 participants was required. To account for potential exclusions and ensure adequate power, 45 participants were recruited (15 per group). All recruited participants completed the study and were included in the final analysis.

Unless otherwise stated, all data are presented as mean ± standard deviation (SD), with individual data points shown in all figures. Normality of distributions was assessed and confirmed using the Shapiro–Wilk test. Resistance training characteristics between BB-NAT and BB-USE were compared using independent two-tailed *t* tests. All other variables were analyzed using one-way ANOVA. When a significant overall effect was detected, Bonferroni-adjusted *post hoc* tests were applied. Statistical significance was set at *P* ≤ 0.05. Data were analyzed using SPSS (version 28.0, IBM Corp.) or GraphPad Prism version 8.2.1 (GraphPad Software Inc., La Jolla, CA).

## RESULTS

### Participants’ characteristics

Participants’ characteristics are presented in Table 1. Age and height did not differ between groups. Total BM was significantly greater in BB-USE compared with BB-NAT and CON (both *P* < 0.001), with no difference between BB-NAT and CON (*P* = 0.246). Consequently, body mass index was also significantly greater in BB-USE than in the other groups (both *P* < 0.001). Total body fat mass and percentage were significantly lower in both bodybuilder groups compared with CON (*P* < 0.005), with no differences between BB-NAT and BB-USE (*P* = 1.000). Bone mineral content showed a significant overall group effect, but Bonferroni-adjusted pairwise comparisons did not identify significant differences between individual groups (all *P* > 0.05). Bone mineral density did not differ between groups. Basal metabolic rate was significantly higher in the BB-USE than in both BB-NAT and CON (both *P* < 0.001), with no difference between BB-NAT and CON (*P* = 0.232). Resting heart rate was also significantly higher in BB-USE than in the other groups (both *P* < 0.001), with no difference between BB-NAT and CON (*P* = 1.000). Diastolic blood pressure did not differ between groups, whereas systolic blood pressure was higher in BB-USE compared with CON (*P* = 0.009).

**TABLE 1.** Participants’ characteristics.

	CON	BB-NAT	BB-USE	*P*
Age (yr)	29 ± 6	30 ± 5	31 ± 4	0.816
Height (m)	1.81 ± 0.06	1.80 ± 0.06	1.84 ± 0.03	0.098
Total BM (kg)	80.9 ± 8.7	86.1 ± 7.0	102.4 ± 8.3^a^^,^^b^	<0.001
Total body fat mass (kg)	14.7 ± 4.5	10.0 ± 2.0^a^	10.9 ± 2.7^a^	<0.001
Total body fat percentage (%)	17.8 ± 3.8	11.7 ± 2.2^a^	10.7 ± 2.6^a^	<0.001
Body mass index (kg/m^2^)	24.5 ± 2.6	26.3 ± 1.8	29.9 ± 1.9^a^^,^^b^	<0.001
Bone mineral content (kg)	3.4 ± 0.3	3.4 ± 0.4	3.7 ± 0.2	0.037
Bone mineral density (g/cm^2^)	1.41 ± 0.09	1.40 ± 0.11	1.43 ± 0.06	0.671
Basal metabolic rate (kcal/d)	1806 ± 145	1957 ± 194	2683 ± 348^a^^,^^b^	<0.001
Resting heart rate (bpm)	58 ± 8	58 ± 7	71 ± 13^a^^,^^b^	<0.001
Systolic blood pressure (mmHg)	117 ± 7	123 ± 11	130 ± 13^a^	0.011
Diastolic blood pressure (mmHg)	68 ± 7	64 ± 6	68 ± 8	0.180

Values represent means ± SD, *n* = 15 per group.

a Significantly different from CON.

b Significantly different from BB-NAT.

### Resistance exercise training characteristics

There were no differences between the BB-NAT and BB-USE groups in total training duration (11 ± 5 vs 10 ± 4 yr, respectively; *P* = 0.388) or weekly training frequency (6 ± 1 vs 6 ± 1 d/wk; *P* = 0.788). However, the BB-NAT group spent more hours per week in the gym than the BB-USE group (10 ± 3 vs 8 ± 2 h/wk; *P* = 0.032) and had longer average training session durations (106 ± 28 vs 84 ± 12 min/session; *P* = 0.015). In addition, the BB-NAT group trained each muscle group more frequently per week than the BB-USE group (2.5 ± 1.2 vs 1.7 ± 0.4 d/wk; *P* = 0.043). All bodybuilders reported that their current training regimen had been followed consistently over the past months to years without major changes.

### Habitual dietary food intake

Daily energy and macronutrient intakes for all groups are presented in Table 2. Absolute energy intake was significantly higher in BB-USE than CON (*P* = 0.029), whereas relative energy intake did not differ between groups. Macronutrient distribution showed clear differences, with both BB-NAT and BB-USE groups deriving a greater proportion of their energy intake from protein and a smaller proportion from fat compared with CON (*P* < 0.05). In addition, the BB-USE group derived a lower proportion of energy from fat than the BB-NAT group (*P* = 0.022). Carbohydrate intake contributed a similar proportion of total energy intake across all groups.

**TABLE 2.** Habitual dietary intake of study participants.

	CON	BB-NAT	BB-USE	*P*
Energy intake				
Energy (kcal/d)	2602 ± 461	2916 ± 806	3393 ± 907^a^	0.032
Energy (kcal·kg BM^-1^·d^-1^)	33 ± 6	34 ± 9	34 ± 8	0.947
Macronutrient distribution				
Protein (En%/d)	17 ± 2	30 ± 6^a^	36 ± 9^a^	<0.001
Carbohydrate (En%/d)	47 ± 5	42 ± 5	42 ± 10	0.082
Fat (En%/d)	36 ± 4	29 ± 7^a^	23 ± 5^a^^,^^b^	<0.001
Macronutrient intake				
Protein (g/d)	110 ± 17	213 ± 57^a^	288 ± 47^a^^,^^b^	<0.001
Protein (g·kg BM^-1^·d^-1^)	1.4 ± 0.3	2.5 ± 0.6^a^	2.9 ± 0.4^a^	<0.001
Protein (g·kg LBM^-1^·d^-1^)	1.8 ± 0.3	2.9 ± 0.7^a^	3.3 ± 0.5^a^	<0.001
Carbohydrate (g/d)	306 ± 62	305 ± 102	369 ± 167	0.283
Carbohydrate (g·kg BM^-1^·d^-1^)	3.9 ± 0.8	3.6 ± 1.1	3.6 ± 1.6	0.792
Fat (g/d)	104 ± 24	94 ± 39	85 ± 26	0.281
Fat (g·kg BM^-1^·d^-1^)	1.3 ± 0.3	1.1 ± 0.4	0.8 ± 0.3^a^	0.005

Values represent means ± SD, *n* = 15 per group. Participants’ habitual energy, protein, carbohydrate, and fat intakes were calculated from a 7-d dietary record. Data were analyzed using a one-way ANOVA. Bonferroni post hoc tests were applied when overall effects were significant.

a Significantly different from CON.

b Significantly different from BB-NAT.

En%, percentage of energy; kg LBM, kilogram lean body mass.

For total macronutrient intake, both bodybuilding groups consumed significantly more protein, in absolute terms as well as expressed relative to (lean) BM, compared with CON, with BB-USE reporting the highest absolute intake (all *P* < 0.001). Relative protein intake (to lean or total BM) did not differ between the two bodybuilding groups (*P* > 0.05). Absolute carbohydrate and fat intake did not differ between groups (*P* > 0.05). However, when adjusted for BM, BB-USE consumed significantly less fat than CON (*P* = 0.004).

### Supplement use

A wide variety of supplements were reported among participants, although most were used by only a single individual (e.g., BCAA in only one natural bodybuilder). Therefore, only the most commonly ingested supplements are presented here. Overall, 7/15 control participants reported no supplement use, compared with 1/15 in the natural bodybuilder group, whereas all APED-using bodybuilders reported habitual supplement intake.

The most frequently used supplements included omega-3 fatty acids (2/15 in CON, 10/15 in BB-NAT, and 8/15 in BB-USE), vitamin D3 (1/15 in CON, 10/15 in BB-NAT, and 7/15 in BB-USE), creatine (1/15 in CON, 9/15 in BB-NAT, and 6/15 in BB-USE), multivitamins (2/15 in CON, 5/15 in BB-NAT, and 9/15 in BB-USE), whey protein (2/15 in CON, 5/15 in BB-NAT, and 7/15 in BB-USE), and magnesium (1/15 in CON, 6/15 in BB-NAT, and 7/15 in BB-USE). Other supplements such as vitamin C and zinc were also used by multiple participants but less frequently.

### Appearance- and performance-enhancing drugs

Participants in the BB-USE group reported current use of a wide range of APED and related pharmacological agents, as detailed in Table 3. Nearly all users (93.3%) used exogenous testosterone esters, such as testosterone enanthate or cypionate. The mean self-reported duration of APED use was 6 ± 3 yr, with an average weekly dosage of testosterone esters (*n* = 14) of 435 ± 257 mg (range: 131–750 mg/wk). The total weekly androgen dosage (*n* = 15) averaged 1019 ± 760 mg (range: 58–2100 mg/wk).

**TABLE 3.** Self-reported appearance- and performance-enhancing drug use.

Substance type	Substances used	Number of participants	Percentage (%)
Testosterone esters	Testosterone enanthate, testosterone cypionate, testosterone propionate	14/15	93.3
Testosterone derivatives	Boldenone undecylenate, dihydroboldenone (1-testosterone)	3/15	20.0
DHT derivatives	Methenolone enanthate, drostanolone enanthate, drostanolone propionate, oxandrolone, stanozolol, oxymetholone	9/15	60
19-Nortestosterone derivatives	Nandrolone decanoate, nandrolone phenylpropionate, trenbolone enanthate, trenbolone acetate, trenbolone hexahydrobenzylcarbonate	7/15	46.7
Aromatase inhibitors	Anastrozole, exemestane, letrozole	5/15	33.3
Prolactin inhibitor	Cabergoline	1/15	6.7
Peptide hormones	HGH, insulin	3/15	20
LH mimetic	hCG	1/15	6.7
GH secretagogue	Ibutamoren (MK-677)	1/15	6.7
Thyroid hormones	Liothyronine (T3), levothyroxine (T4)	3/15	20
Sympathomimetic amine	Clenbuterol	3/15	20

Self-reported use of APED. Substances categorized by pharmacological class, with specific agents listed. Values represent the number and percentage of participants (*n* = 15) who reported current use of each category.

DHT, dihydrotestosterone; LH, Luteinizing hormone; HGH, human growth hormone; hCG, human chorionic gonadotropin.

### Lean body mass

**FIGURE 1:**
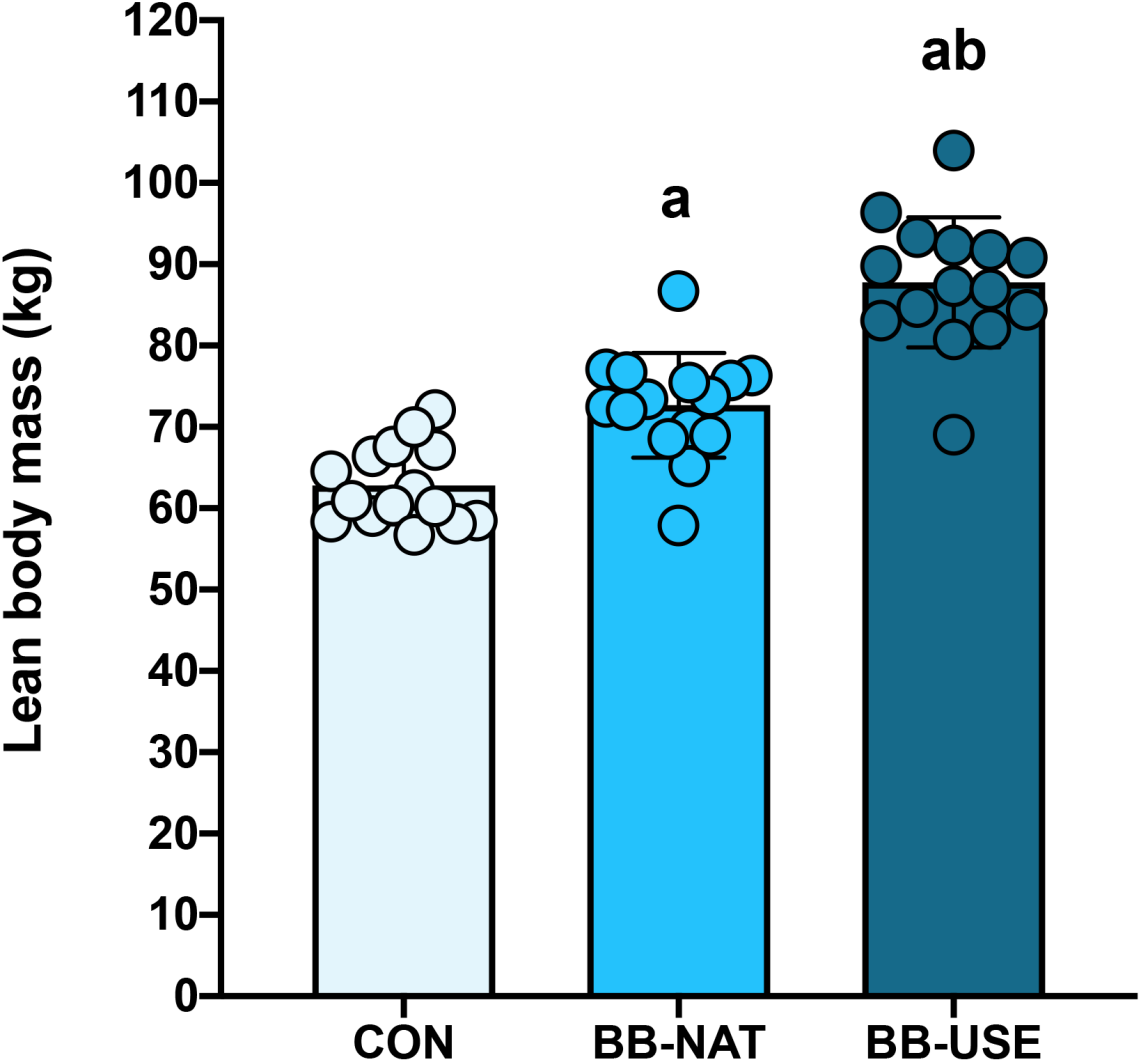
DXA-derived total lean BM (defined here as total BM minus fat mass and bone mineral content) in CON, BB-NAT, and BB-USE. Values represent means ± SD, and dots represent individual values, *n* = 15 per group. Data were analyzed using a one-way ANOVA with Bonferroni *post hoc* correction. ^a^Significantly different from CON. ^b^Significantly different from BB-NAT.

Total lean BM, assessed by DXA, differed significantly between groups (all *P* < 0.001). BB-USE showed the highest lean BM (87.8 ± 8.0 kg), followed by BB-NAT (72.7 ± 6.4 kg), while CON had the lowest lean BM (62.8 ± 4.8 kg; Figure 1).

### Skeletal muscle volume

**FIGURE 2:**
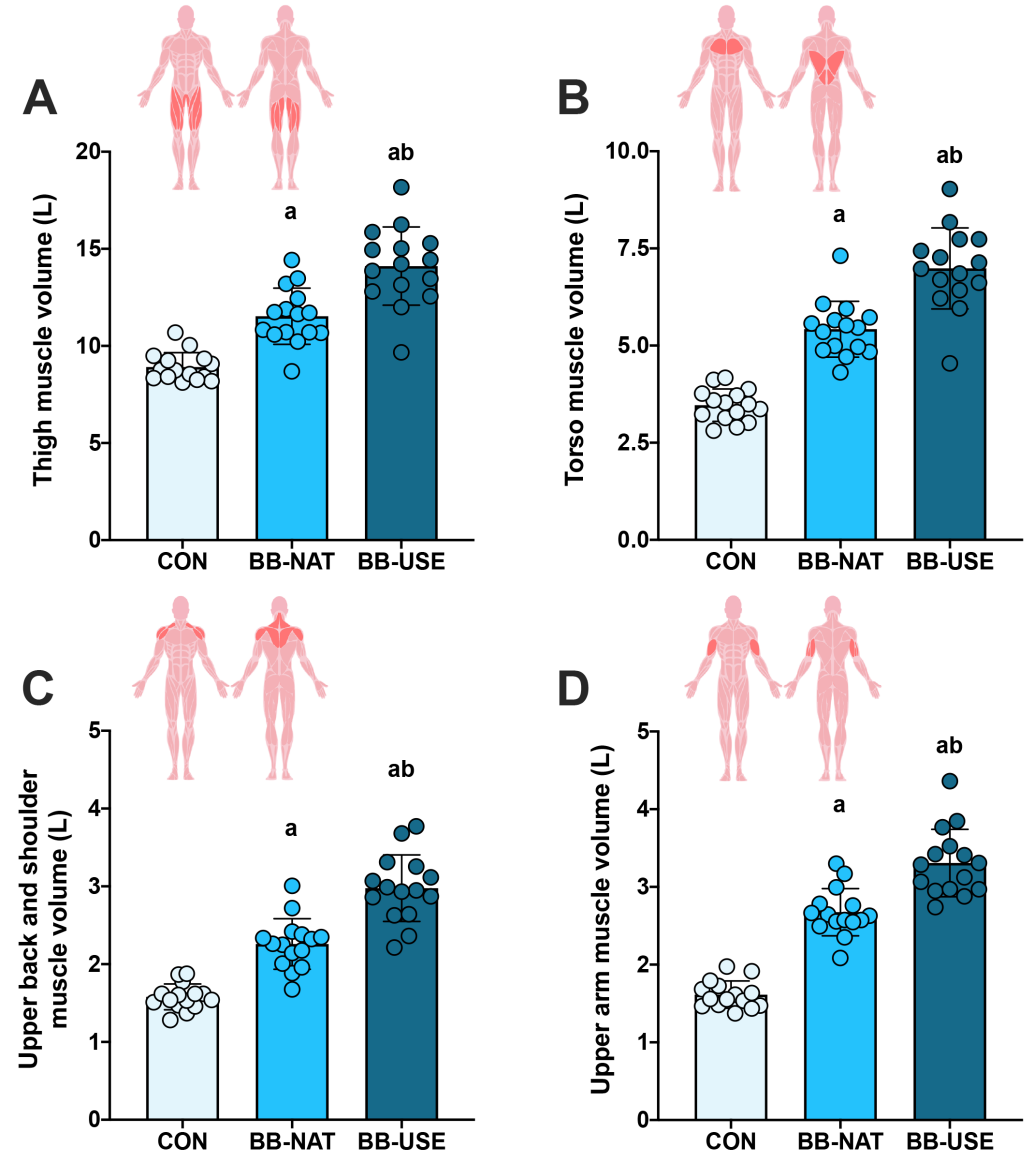
MRI-derived total thigh muscle volume (A), torso muscle volume (B), upper back and shoulder muscle volume (C), and upper arm muscle volume (D) in CON, BB-NAT, and BB-USE. Muscle regions were categorized as follows: Thigh: *quadriceps femoris, hamstrings, adductors*; Torso: *pectoralis major, latissimus dorsi/teres major, erector spinae* (Th1–sacrum); Upper back/shoulder: *trapezius, deltoid*; Upper arm: *biceps brachii, triceps brachii*. Values represent means ± SD and dots represent individual values, *n* = 15 per group. Data were analyzed using a one-way ANOVA with Bonferroni *post hoc* correction. ^a^Significantly different from CON. ^b^Significantly different from BB-NAT.

Total skeletal muscle volume (sum of left and right sides) of the thighs, torso, upper back and shoulders, and upper arms is shown in Figure 2. Volumes differed significantly between groups (all *P* < 0.001), with BB-USE exhibiting the greatest muscle volumes, followed by BB-NAT, and CON showing the lowest.

Average muscle volumes of the individual muscle groups are presented in Table 4. No differences were observed between left and right sides; therefore, values are reported as the mean of a single side. The results show a consistent pattern across all muscles, with BB-USE having the greatest volumes, followed by BB-NAT, and CON showing the lowest (all *P* < 0.010).

**TABLE 4.** Muscle volumes of major muscle groups.

Volume (cm^3^)	CON	BB-NAT	BB-USE	*P*
Thigh				
Quadriceps femoris	2364 ± 250	3069 ± 343^a^	3829 ± 538^a^^,^^b^	<0.001
Hamstring	903 ± 65	1184 ± 184^a^	1418 ± 197^a^^,^^b^	<0.001
Adductor	1189 ± 119	1511 ± 268^a^	1809 ± 304^a^^,^^b^	<0.001
Torso				
Pectoralis major	458 ± 78	832 ± 132^a^	1029 ± 152^a^^,^^b^	<0.001
Latissimus dorsi/Teres major	492 ± 65	899 ± 156^a^	1203 ± 225^a^^,^^b^	<0.001
Erector spinae (Th1-sacrum)	782 ± 131	979 ± 120^a^	1262 ± 235^a^^,^^b^	<0.001
Upper back/shoulder				
Trapezius	275 ± 38	383 ± 46^a^	530 ± 84^a^^,^^b^	<0.001
Deltoid	514 ± 54	747 ± 121^a^	959 ± 143^a^^,^^b^	<0.001
Upper arm				
Biceps brachii	269 ± 30	442 ± 54^a^	541 ± 80^a^^,^^b^	<0.001
Triceps brachii	538 ± 61	896 ± 107^a^	1113 ± 151^a^^,^^b^	<0.001

For all muscles, values represent a single side (mean of the left and right side). Values represent means ± SD, *n* = 15 per group.

a Significantly different from CON.

b Significantly different from BB-NAT.

Data were analyzed using a one-way ANOVA with Bonferroni *post hoc* correction.

### Visceral organ volume

**FIGURE 3:**
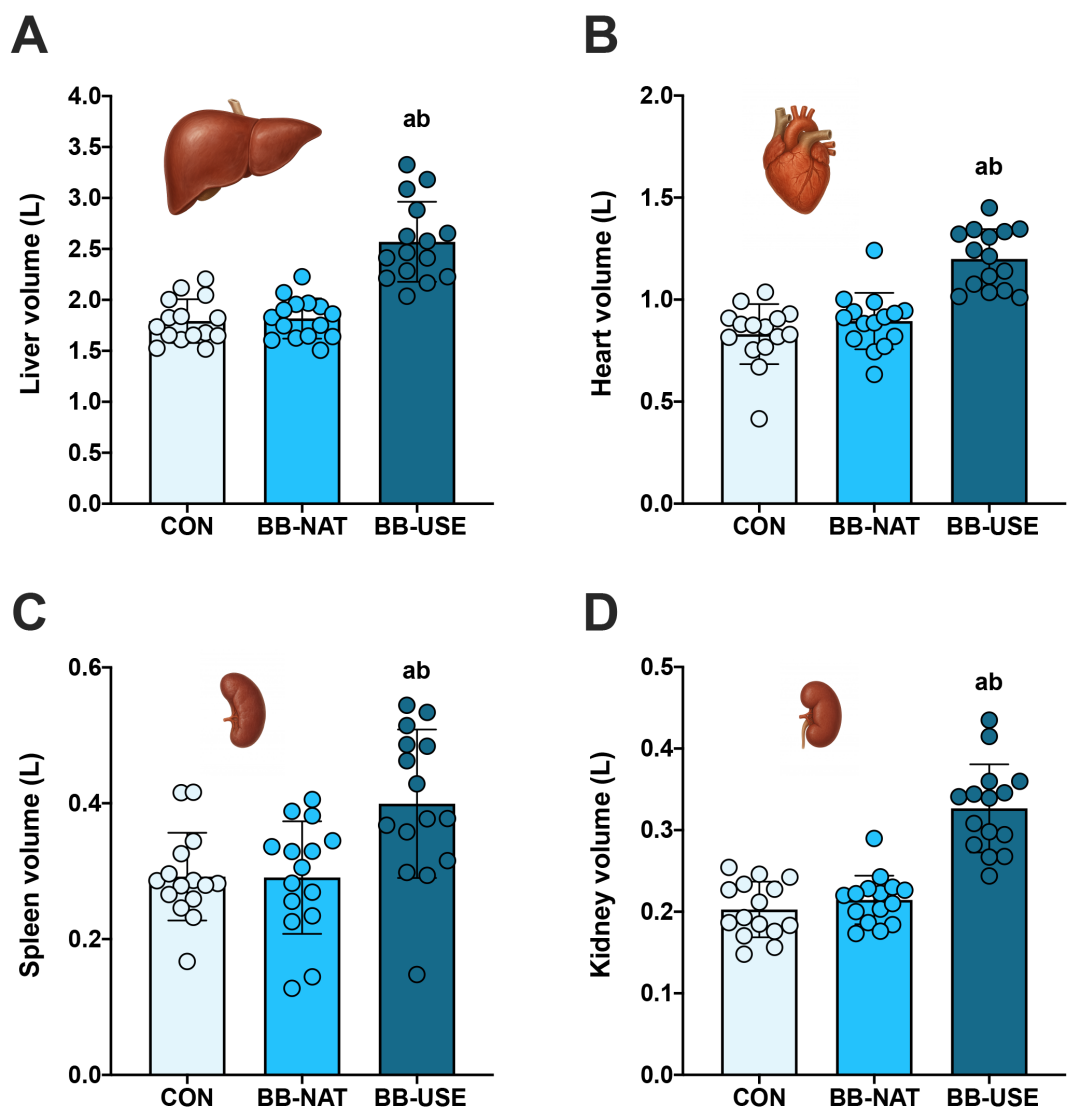
MRI-derived liver volume (A), heart volume (B), spleen volume (C), and kidney volume (D) in CON, BB-NAT, and BB-USE. For kidney volume, the average (left and right combined) values are depicted. Values represent means ± SD and dots represent individual values, *n* = 15 per group. Data were analyzed using a one-way ANOVA with Bonferroni post hoc correction. ^a^Significantly different from CON. ^b^Significantly different from BB-NAT.

Volumes of the liver, heart, spleen, and kidneys are shown in Figure 3. All organ volumes were significantly greater in BB-USE compared with BB-NAT, and CON (all *P* < 0.005), with no differences between BB-NAT and CON (all *P* > 0.05). The same pattern was observed when kidney volumes were analyzed separately, with BB-USE having significantly greater left (337 ± 61 mL) and right kidney volumes (317 ± 52 mL) compared with BB-NAT (219 ± 38 mL and 210 ± 27 mL, respectively) and CON (215 ± 55 mL and 190 ± 37 mL, respectively; *P* < 0.001).

### Scatterplots

**FIGURE 4:**
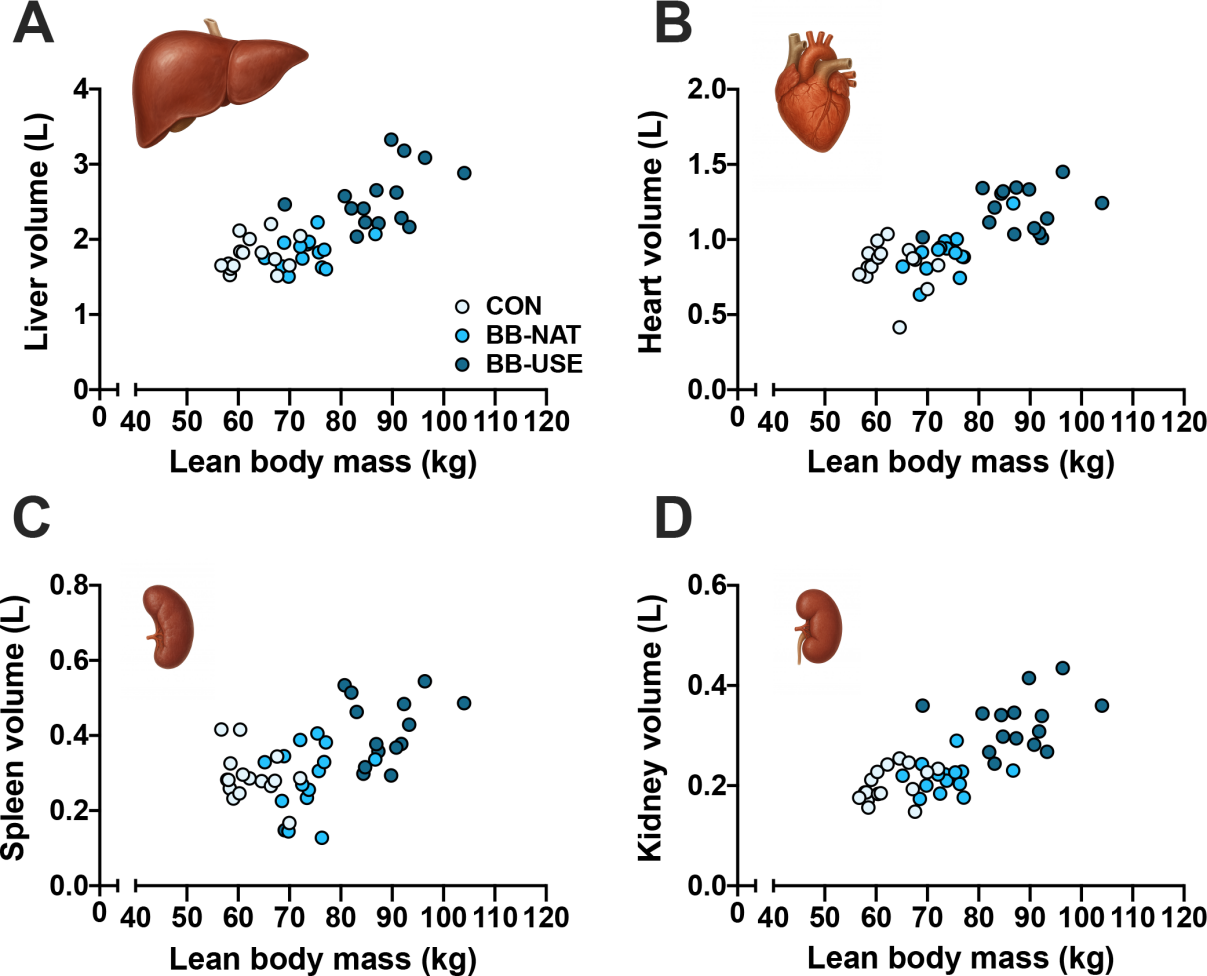
Scatterplots of lean BM versus organ volumes in CON, BB-NAT, and BB-USE.

Scatterplots of lean BM versus organ volumes are shown in Figure 4 and are presented for descriptive purposes to illustrate the relationship between lean BM and visceral organ volumes across groups. Although BB-NAT had greater lean BM than CON, the two groups showed largely overlapping organ volumes, whereas BB-USE showed a clear shift toward greater lean BM and organ volumes.

## DISCUSSION

In the present study, we observed that only bodybuilders using APED showed enlarged visceral organ volumes when compared with both natural bodybuilders and controls. The natural bodybuilders, despite a high-protein intake, did not show greater visceral organ volumes when compared with age-matched controls. Therefore, the greater lean mass in the natural bodybuilders compared with controls was attributed to greater skeletal muscle mass. Bodybuilders using APED showed greater lean BM when compared with both natural bodybuilders and controls, which was attributed to both substantially enlarged visceral organ volumes and greater skeletal muscle mass.

Bodybuilders typically engage in progressive resistance exercise training and adopt a high-protein intake with the goal of maximizing skeletal muscle mass while minimizing body fat. In the present study, the natural bodybuilders we recruited had trained for an average of 11 ± 5 yr and reported training 6 ± 1 d/wk. Their habitual protein intake was three times higher than the estimated requirement for healthy adults (36), ~60% higher than the intake estimated as sufficient to maximize muscle growth (3,37), and ~80% higher than that of age-matched controls (Table 2). As expected, compared with the controls, the natural bodybuilders had a lower body fat percentage and substantially greater lean BM. Lean BM is often used as a proxy for skeletal muscle (38). However, it is important to note that lean BM comprises not only skeletal muscle but also other tissues, such as internal organs (32). Distinguishing between these components is important, as previous studies have suggested that prolonged resistance exercise training and high-protein intake may not only increase skeletal muscle mass but also augment visceral organ mass (14,15). Therefore, the greater lean BM typically observed in natural bodybuilders could reflect increases in both skeletal muscle and visceral organ mass. In the present study, MRI was applied to assess skeletal muscle volumes of major muscle groups and visceral organ volumes (liver, heart, spleen, and kidneys) in natural bodybuilders and controls. As expected, all skeletal muscle volumes were substantially greater in the natural bodybuilders compared with the age-matched controls, with the largest relative differences observed in the upper body muscles (Figure 2 and Table 4). In contrast, visceral organ volumes did not differ between groups (Figure 3). Therefore, these findings strongly suggest that the greater lean BM in natural bodybuilders reflects skeletal muscle hypertrophy without accompanying visceral organ enlargement.

Beyond structured resistance exercise training and high-protein intake, many bodybuilders also use APED to further augment skeletal muscle mass while maintaining low body fat (39–41). In the present study, APED-using bodybuilders reported a similar training duration and frequency compared with the natural bodybuilders. Their relative habitual protein intake was also not significantly different from the natural bodybuilders (Table 2). By contrast, APED users reported long-term drug use, with an average duration of 6 ± 3 yr (Table 3). As expected, their total lean BM was substantially greater than that of natural bodybuilders and controls (Figure 1). As with the natural bodybuilders and controls, MRI was used to assess skeletal muscle and visceral organ volumes in the APED-using bodybuilders to further characterize the composition of their lean BM. Analysis of skeletal muscle volumes indicated that the greater lean BM in APED-using bodybuilders was at least partly attributable to increased muscle volumes (Figure 2 and Table 4). Skeletal muscle volumes were ~20%–40% higher in APED-using bodybuilders compared with natural bodybuilders, with the largest relative difference observed in the trapezius muscle (Table 4). The latter is not surprising given that the trapezius muscle, in particular, expresses high levels of androgen receptors (42). As APED use has also been implicated in visceral organ enlargement (24,25,43–47), we assessed visceral organ volumes in APED-using bodybuilders. In contrast to natural bodybuilders (who did not differ from controls), APED users displayed substantially greater organ volumes, by approximately 35%–40% for the liver, heart, and spleen, and more than 50% for the kidneys (Figure 4). Consequently, visceral organ enlargement contributes to the greater lean BM observed in APED-using bodybuilders. Since relative dietary protein intake and main training characteristics did not differ between the bodybuilding groups, these findings strongly suggest that visceral organ hypertrophy is attributed to the use of APED.

Visceral organ enlargement, medically referred to as visceromegaly, has been described for over two decades within the bodybuilding community. However, this phenomenon has largely been speculative, often inferred from the visibly distended abdomens of some athletes. Such abdominal distention may also arise from other factors, including (transient) gastrointestinal distention owing to high food and fluid intake volumes, increased visceral fat accumulation, or structural changes in the abdominal wall (e.g., connective tissue remodeling leading to reduced fascial stiffness and diminished abdominal wall tension) (24). However, these factors are unlikely to explain the persistent abdominal protrusion often observed in lean, hypohydrated bodybuilders during competition, suggesting that true visceral organ enlargement may play a key role. Nonetheless, available evidence for visceromegaly has remained largely anecdotal and limited to isolated case reports and (publicly available) autopsy findings (28,44,45,48,49). The present study provides clear quantitative evidence that visceromegaly is indeed present in bodybuilders using APED, with a consistent pattern of enlarged visceral organ volumes across all individuals and organs assessed. In addition to contributing to the characteristic “bubble gut” appearance, the health implications of this enlargement need to be better understood.

Enlargement of metabolically active organs such as the liver and kidneys may not simply reflect volume changes but are likely also indicative of altered organ function. Liver and kidney abnormalities (e.g., elevated enzymes, steatosis, cholestasis, carcinomas, and even organ failure) have been documented in some APED-using bodybuilders (23,46,48,50–52). However, consistent evidence of clinically relevant dysfunction and damage in APED-using bodybuilders is lacking (47,53). Beyond the liver and kidneys, the heart warrants particular attention. Although increased cardiac mass can represent a physiological adaptation to resistance training, excessive or disproportionate enlargement may have adverse consequences. The higher resting heart rate and systolic blood pressure observed in APED-using bodybuilders in the present study (Table 1), although not consistently reported in the literature (23,54), aligns with previous findings in APED users (25,55) and may indicate early signs of altered cardiovascular function. Chronic exposure to APED has been associated with cardiovascular dysfunction and an increased risk of sudden cardiac death (27,28,54,55). Therefore, the observed cardiac hypertrophy in these athletes may not represent a benign or purely training-induced adaptation. Overall, it remains unclear whether the observed organ enlargement across the liver, kidneys, spleen, and heart represents pathological remodeling owing to organ stress or damage, an adaptive response to elevated metabolic activity and energy turnover (Tables 1 and 3), or a combination of both. Nonetheless, our findings provide an important foundation for future research examining the functional and clinical consequences of the observed increase in organ sizes in bodybuilders using APED, given that our data imply that organ enlargement is not attributed to a high-protein intake.

Despite ongoing caution surrounding very high-protein intakes, there is little evidence to suggest that chronic consumption of protein-rich diets causes organ dysfunction in otherwise healthy individuals (16,18). While the protein intakes observed in the present study can be considered extremely high and likely unnecessary to support (further) muscle gains (3,37), it is important to note that such high absolute intakes are not uncommon in bodybuilding or other sports (1,56,57). Such high absolute intakes can even occur when protein contributes only a moderate proportion of total energy intake. For example, endurance athletes easily reach daily energy expenditures of ≥25 MJ (~6000 kcal) during intense and prolonged exercise (57,58). With protein contributing ~15% of total energy intake, protein intake would reach well over 3 g/kg/d in these athletes. Taken together, our findings indicate that athletes do not need to be concerned about developing visceromegaly as a result of high daily total protein intake. However, they should be aware that there are unlikely benefits to expect from ingesting large amounts of protein (>1.5 g/kg/d) with regard to muscle tissue conditioning.

Several factors should be considered when interpreting these findings. First, although APED use was clearly associated with substantially greater muscle and visceral organ volumes, the cross-sectional design precludes conclusions about causality. Second, the APED-using bodybuilders reported using a heterogeneous mix of substances, doses, and administration routes, preventing determination of which specific agents may have contributed to the enlarged organ volumes. Third, APED use may cause water retention, meaning estimates of lean body, muscle, and visceral organ mass may partially reflect an increase in body water as part of an increase in tissue mass. Finally, although we assessed competitive natural bodybuilders competing in natural divisions, who are subject to in-competition and random drug testing, blood samples were not collected to assess androgen hormone concentrations or other pharmacological substances.

In short, the greater lean BM in bodybuilders using APED is attributed to greater skeletal muscle mass as well as substantially enlarged visceral organ mass. Greater visceral organ volumes are not apparent in natural bodybuilders despite a very high habitual protein intake. We conclude that visceromegaly in bodybuilders cannot be attributed to a high-protein intake but is secondary to the use of APED.

No conflicts of interest were disclosed.

The authors like to acknowledge the enthusiastic support of the participants who volunteered to participate in this experiment. The authors declare that they have no conflicts of interest regarding this work and the publication of this article. The results of the study are presented clearly, honestly, and without fabrication, falsification, or inappropriate data manipulation. The results of the present study do not constitute endorsement by the American College of Sports Medicine. C.J.F., J.T., and L.J.C.L. conceived and designed the research. C.J.F. and B.B. generated and collected data. P.W., O.D.L., and J.J.P. provided methodological input and resources. C.J.F., B.B., and S.Y. analyzed the data. C.J.F. and L.J.C.L. interpreted the results. C.J.F. prepared the figures. C.J.F. drafted the manuscript. C.J.F. and L.J.C.L. edited and revised the manuscript. All authors approved the final version submitted for publication. All data generated and analyzed during this study are available from the corresponding author on reasonable request. C.J.F. and L.J.C.L. obtained funding from the Stimuleringsfonds FHML, Maastricht, the Netherlands. This funding did not influence the study design, data analysis, or interpretation of results.
